# Effects of zearalenone on ovarian development of prepubertal gilts through growth hormone axis

**DOI:** 10.3389/fvets.2022.950063

**Published:** 2022-08-03

**Authors:** Fengyang Wu, Lijie Gao, Fei Li, Jia Cui, Xinyu Yang, Yanhua Liu, Saijuan Chen, Baojiang Chen

**Affiliations:** ^1^College of Animal Science and Technology, Hebei Agricultural University, Baoding, China; ^2^Hebei Provincial Animal Husbandry Station, Shijiazhuang, China; ^3^Mountainous Area Research Institute of Hebei Province, Hebei Agricultural University, Baoding, China; ^4^Agricultural Technology Innovation Center in Mountainous Areas of Hebei Province, Baoding, China

**Keywords:** zearalenone, prepubertal gilts, ovary, growth hormone-releasing hormone, growth hormone, growth hormone receptor

## Abstract

This experiment aimed to establish the effects of zearalenone (ZEN) on ovarian development in prepubertal gilts through the growth hormone axis [growth hormone-releasing hormone (GHRH) / growth hormone (GH) / growth hormone receptor (GHR)]. In a 40-day experiment, 48 Landrace × Yorkshire crossbred prepubertal gilts were randomly allocated to four dietary treatments, including a basal diet supplemented with 0 (control), 400 (T1), 800 (T2), and 1,600 (T3) μg/kg ZEN. The ovary index of T2 (*P* = 0.058) and T3 (*P* = 0.065) increased compared to the control group. Besides, histopathological examination revealed that ZEN promoted the development of ovaries and follicles. The GHR content, relative expression levels of *GHR*, janus activated kinase 2 (*JAK2*) mRNA, and mean optical density of GHR in the ovaries of prepubertal gilts in the T2 experimental group increased significantly at *P* < 0.05 compared to the control group. The T3 group had significantly higher GHR content, relative *JAK2* expression levels, and signal transducer and activator of transcriptions 3 (*STAT3*) mRNA. In conclusion, ZEN enhances the biological effect of GH, promotes the development of the ovary (follicle), and exerts reproductive toxicity by increasing the expression level of *GHR, JAK2*, and *STAT3* mRNA ovary and immune intensity of GHR protein.

## Introduction

Zearalenone (ZEN) is a mycotoxin mainly produced by *Fusarium* and has estrogenic activity and various toxic effects (1). This mycotoxin is widely found in feedstuff and compound feeds of pigs and ranks among the most common and harmful mycotoxins in pig production (2, 3). Besides, ZEN affects the reproductive and immune performance of pigs in different growth stages. In a previous study done by Gruber-Dorninger et al. (4) from 2008 to 2017, 61,413 feedstuff and feed samples were collected worldwide. The test results showed a ZEN contamination rate of 88%, a median of 55 μg/kg, and the highest pollution value of 105 mg/kg.

Ovary is an important reproductive organ of sows, and its growth, development, and functional status directly affects the reproductive performance and breeding value of sows (5). Numerous studies have shown that ZEN affects the development of sows' ovaries and exert reproductive toxicity. According to Dai et al. (6), 1.04 mg/kg of ZEN promotes ghrelin gene expression in a piglet ovary and accelerates ovarian follicular depletion *via* massive follicular activation. In addition, Yang et al. (7) reported that ZEN activates estrogen receptors (ERs)/glycogen synthase kinase (GSK)-3β-dependent Wnt-1/β-catenin signaling pathway and promotes the development of the porcine ovary. Maternal ZEN exposure affects the ovarian development of offspring through breast milk (8). ZEN affects ovarian development in many ways. However, few reports indicate how it affects ovary development through the growth hormone axis (GHRH/GH/GHR). The growth hormone-releasing hormone (GHRH) peptide hormone is secreted by the hypothalamus, which stimulates the pituitary gland to secrete the growth hormone (GH) (9). The growth hormone receptor (GHR) is a single-stranded transmembrane glycoprotein, which regulates the target organs growth by signaling autocrine or paracrine through GH-specific binding (10). According to Zhou et al. (11) GHR is distributed in the ovaries and uterus of pigs, while ZEN increases the relative weight of the uteri and thickness of the myometrium and endometrium of the pig by regulating the GHR expression.

In women, the non-growing population of follicles that constitutes the ovary reserve function is determined at birth (12). This reserve of the dormant, primordial follicle and the mechanism controlling their selective activation is closely related to the women reproductive potential. On the other hand, when the ovary develops too fast, the excessive induction of primordial follicle activation leads to ovarian dysfunction. Previously, ZEN and its derivatives have been detected in various foods such as infant food supplements, bread, and dairy products (13, 14). Besides, ZEN exposure may lead to numerous diseases such as premature thelarche, idiopathic central precocious puberty, hormonal disruption, ovarian cancer and cervical cancer (15, 16).

The ZEN contamination causes economic losses in the pig industry and poses a potential threat to food safety and human health. Currently, the ZEN mechanism on ovary and follicle development has not been fully elucidated. Therefore, this experiment aimed to study the ZEN effects on ovary development of the prepubertal gilts through the growth hormone axis (GHRH/GH/GHR). The findings reported here in will provide a reference for exploring the toxic mechanism of ZEN.

## Materials and methods

### Experimental diet

ZEN with a guaranteed purity ≥98% was purchased from Triplebond (Guelph, Ontario, Canada). Prepubertal gilts were fed with corn-soybean meal diet with no antibiotics and mildew remover. The basic diet was prepared before the experiment using National Research Council (NRC) (2012) protocol; its composition and nutrition levels are shown in Table 1.

**Table 1:** Composition and nutrient levels of the basal diet (air-dry basis).

**Items**	**Content (%)**
**Ingredients**
Corn	65.00
Soybean meal	19.00
Wheat bran	12.00
Premix^a^	4.00
Total	100.00
**Nutrient levels**
Digestible energy^b^, (MJ/kg)	14.06
Dry matter	87.36
Crude protein	20.34
Crude fiber	2.18
Calcium	0.88
Phosphorus	0.64
Available phosphorus	0.46
Lysine	1.22
Methionine	0.41
Threonine	0.81

a *Premix provides the following components (per kg of the diet): VA 360 000 IU, VD 3 60 000 IU, VE 375 mg, VK3 120 mg, VB1 50 mg, VB2 180 mg, VB6 90 mg, VB12 0.63 mg, niacin 1,000 mg, pantothenic acid 630 mg, biotin 12 mg, choline 19 g, folic acid 100 mg, Salt 75 g, lysine 15 g, Fe (as ferrous sulfate) 3 g, Cu (as copper sulfate) 0.375 g, Mn (as manganese sulfate) 1.047 g, Zn (as zinc sulfate), I (as potassium iodide), Se (as sodium selenite), Cr 6 mg, Ca 155 g and P 35 g*.

b *DE was a calculated value, while the others were measured values*.

Dietary preparation was completed before the experiments. Based on the enzyme linked immunosorbent assay (ELISA), the ZEN contents in the diets of each group were; 52.37, 241.60, 825.20, and 1 634.46 μg/kg, while other mycotoxins (aflatoxin B1, vomitoxin and fumonisin) were not detected. The ZEN, vomitoxin, and aflatoxin B1 kits were purchased from Fender Biotechnology Co., Ltd. (Shenzhen, China), while the fumonisin kits were purchased from Meilian Biotechnology Co., Ltd. (Shanghai, China).

### Experimental design, animal and management

Forty-eight Landrace × Yorkshire crossbred prepubertal gilts (aged 65 ± 3 days, initial body weight 23.20 ± 0.68 kg) were randomly selected and divided into four groups with 12 replicates in each group and one gilt in each replicate. The control group (CON) was fed with a basal diet, while the experimental groups (T1, T2, and T3) were fed with experimental diets obtained by supplementing the basal diet with 200, 800, and 1,600 μg/kg of ZEN. The preliminary trial period was set as 7 days, and the trial period was set as 40 days. Before the experiment, the piggery was cleaned, disinfected, and gilts reared in columns.

The experimental protocols were approved by the Animal Care and Use Committee of Hebei Agriculture University (Baoding, China).

All animal experiments complied with the ARRIVE guidelines and were carried out in accordance with the U.K. Animals (Scientific Procedures) Act, 1986 and associated guidelines, EU Directive 2010/63/EU for animal experiments.

### Measurement indicators and methods

#### Serum and tissue indicators

At the end of the trial period, the test prepubertal gilts were subjected to fasting for 12 h. After that, eight prepubertal gilts were randomly selected from each group, and 15 mL of blood was collected from the anterior vena cava of the sampled prepubertal gilts. The sampled blood was poured into centrifuge tubes, inclined and left to settle for 30 min and then centrifuged at 3000 × g for 10 min to separate the serum. Four prepubertal gilts in each experimental group were randomly selected. The sampled gilts were subjected to euthanasia, and the hypothalamus, pituitary, and ovarian tissues samples were obtained under aseptic conditions, and frozen at−80°C for subsequent tests. Enzyme- ELISA was used to determine the GHRH content in the serum and the GHRH, GH, and GHR contents in the sampled prepubertal gilts tissues. The kit was purchased from Meilian Biotechnology Co., Ltd. (Shanghai, China) and operated according to the instructions.

#### Growth performance and ovarian index

The live prepubertal gilts were weighed before slaughter, while the ovarian tissue was weighed after slaughter. The obtained weight was used to calculate the organ index based on the formula;

Ovarian index (g/kg) = fresh weight of ovary (g) after slaughter/ live weight before slaughter (kg).

#### The ovarian tissue morphology

The ovarian tissues were picked under aseptic conditions and fixed using a 10% neutral formaldehyde fixing solution. The fixed tissues were dehydrated using a full-automatic dehydrator (TSJ-II, Zhongshan, Changzhou, China). After embedding and slicing, the following operations were performed: the fixed tissues were subjected to slice dewaxing. After that, it was put in hematoxylin stain for 15 min and rinsed with tap water for 2 min. Thereafter, hydrochloric acid alcohol differentiation was done for 10 s, rinsed with tap water for 2 min, and put in warm water at 50°C until the solution turned blue. It was then rinsed with tap water for 2 min, and put in 85% alcohol for 4 min. The tissues were then stained with eosin for 4 min, washed with distilled water for 5 s. The tissues were then dehydrated using gradient alcohol, made transparent using xylene, and sealed with neutral gum. The trinocular biological microscope camera system (BA200 digital, motic, Xiamen, China) was used for slice observation and image acquisition. All tissues of each section were observed using a microscopic camera system at X40, and the tissue images were taken at X100 and X400. The type and number of follicles were counted according to Schoevers et al. (17). Follicles were counted and classified as primordial follicles, growing follicles and atretic follicles.

#### Relative gene expression in the tissues

The relative content of mRNA of tissue samples were detected by quantitative real-time PCR (qRT-PCR). The specific primers were designed using primer 6.0 software based on the gene sequences of glyceraldehyde-3-phosphate dehydrogenase (*GAPDH*), *GHRH*, growth hormone releasing hormone receptor (*GHRHR*), *GH, GHR*, janus activated kinase 2 (*JAK2*), and signal transducer and activator of transcriptions 3 (*STAT3*) in the GenBank and were synthesized by Bioengineering Co., Ltd. (Shanghai, China) (Table 2).

**Table 2:** Sequence of primers for real-time PCR.

**Target gene**	**Primer sequence (5' to 3')**	**Product size (bp)**	**GenBank accession No**.
*GAPDH*	Forward: ATGGTGAAGGTCGGAGTGAA	154	NM_001206359.1
	Reverse: CGTGGGTGGAATCATACTGG		
*GHRH*	Forward: TGCCATCTTCACCAACAGCT	111	NM_001195118.1
	Reverse: GCTCCTTGCTCCTGGTTTCT		
*GHRHR*	Forward: CTCCCCCTACTGGTGGATCA	159	L11869.2
	Reverse: GTTGACTTGGAGAGACGCCA		
*GH*	Forward: AGAGGTACTCCATCCAGAAC	228	JF276447.1
	Reverse: GGTATGTCTCAGCCTTGTG		
*GHR*	Forward: CCTCAACTGGACTCTACTG	228	NM_214254.2
	Reverse: ACACGCACTTCATACTCTT		
*JAK2*	Forward: AGACAACACTGGGGAGGTGGT	108	NM_214113.1
	Reverse: TCATGCTGCAAGGATTTAAGGA		
*STAT3*	Forward: GAAAGCAGCAAAGAAGGAGGAG	198	NM_001044580.1
	Reverse: GACCAGCGGAGACACAAGGAT		

*GAPDH, glyceraldehyde-3-phosphate dehydrogenase; GHRH, growth hormone-releasing hormone; GHRHR, growth hormone releasing hormone receptor; GH, growth hormone; GHR, growth hormone receptor; JAK2, Janus activated kinase 2; STAT3, signal transducer and activator of transcriptions 3*.

The total RNA was extracted using 50–100 mg of the ovarian, hypothalamic and hypophyseal samples, respectively, following the Trizol reagent according to the manufacture's instruction (Invitrogen, Carlsbad, USA). The extracted RNA concentration was detected using an RNA concentration meter (Nanodrop Lite, Thermo Fisher Scientific, Massachusetts, USA). A total of 1 μg RNA were used for cDNA synthesis with the kit of HiScript^®^ III RT SuperMix for qPCR (+gDNA wiper) (Number: R323-01, Vazyme, Nanjing, China) based on the manufacture's instruction. The qRT-PCR was performed with CFX96 touch real-time PCR detection system (Bio-Rad, Hercules, CA, USA) using the primers displayed in Table 2. GAPDH was selected as internal control to compare the amplification efficacy. The ChamQ Universal SYBR qPCR Master Mix (Number: Q711-02, Vazyme, Nanjing, China) was used in qRT-PCR. A total of 20 μL qRT-PCR mixture consisted of 10 μL SYBR Green Master Mix, 0.8 μL of forward and reverse primers mix (stock concentration of 10 μmol/L), 2 μL (200 ng) template cDNA and 7.2 μL DNase/RNase Free H_2_O. The heat-cycling conditions of qRT-PCR: 95°C for 5 min, followed by 40 cycles at 95°C for 10 s, 60°C for 30 s of melt curve analysis. The experiment was repeated three times and the target gene expression was calculated by the 2^−ΔΔCt^ method.

#### Immunohistochemical detection

The ovarian tissue was picked under aseptic condition and fixed on using 4% paraformaldehyde fixing solution, and the fixed tissues were dehydrated using a full-automatic dehydrator (TSJ-II, Zhongshan, Changzhou, China). After embedding and slicing, the following operations were carried out: After dewaxing, the sliced tissues were put on a dyeing tank with 3% methanol hydrogen peroxide for 10 min at room temperatures. After that, the tissues were removed and washed three times with PBS, each lasting for 5 min. After washing, the samples slices were immersed in 0.01 M citrate buffer solution (pH = 6.0), heated until it boiled, and allowed to settle. After cooling for 5 min, the same procedure was repeated; washed with PBS twice, each washing step lasting for 5 min. Thereafter, a goat serum was added dropwise to the block solution (Number: zli-9021, ZSBIO, Beijing, China) and allowed to settle at room temperature for 20 min. Besides, primary antibody (GHR, Number: BS-0654R, Bioss, Beijing, China), drops were added and allowed to react overnight at 4°C. After the overnight reaction, a biotinylated secondary antibody (Number: SP-9001, ZSBIO, Beijing, China) was added and allowed to settle at 37°C for 30 min then washed three times with PBS, each step lasting for 5 min. The eighth step was the DAB color development, where the DAB color development kit reagents (Number: K135925C, ZSBIO, Beijing, China) were mixed evenly at room temperature and dropped on the slices to develop the color. The control reaction time under a microscope was set at 2 min, and after that, washed with distilled water. Hematoxylin (Number: LM10N13, J&K Scientific, Beijing, China) was used for light re-dying, followed by dehydration, transparency, and sealing with neutral gum. The microscopic camera system (BA200 digital, motic, Xiamen, China) was used for slice observation and image acquisition. All the tissue slices from each sample were observed at X100, and three visual fields at X400 were selected for microscopic images acquisition. Image-Pro Plus, a 6.0 image analysis system, was used to measure the optical density, area and to calculate the mean optical density (MD) of each image.

### Statistical analysis

The statistical data analysis was done using Excel 2016 and SPSS 20.0 software. One-way analysis of variance (ANOVA) was used to test the significant differences between each group data, while the Duncan method was used for multiple comparisons. A *p* < 0.05 showed a significant difference between the groups, and 0.05 < *P* < 0.10 was considered a tendency.

## Results

### Ovarian index

**Figure 1:**
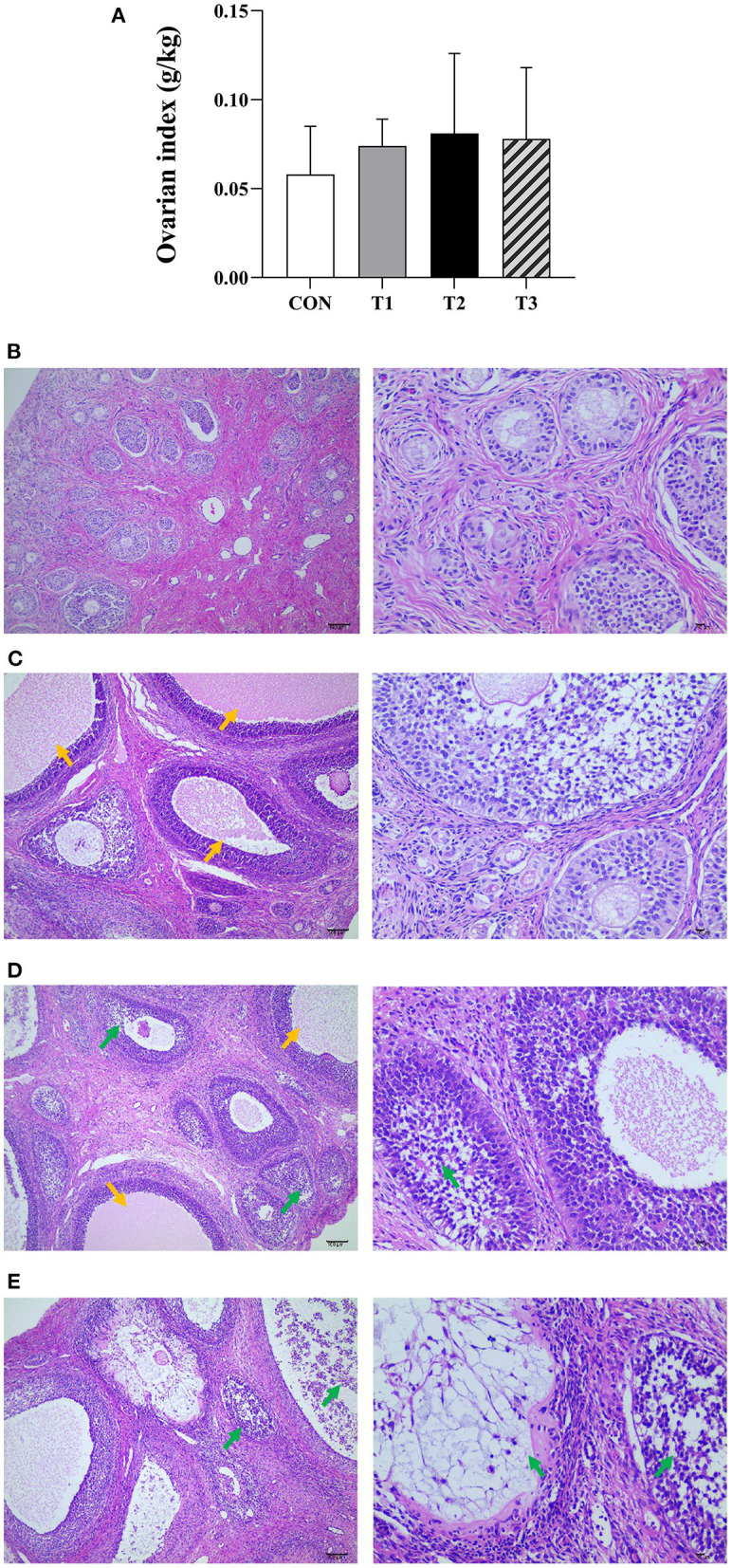
Effects of ZEN on the ovarian index **(A)**, ovarian structure and morphology of prepubertal gilts **(B–E)**. **(B–E)** Images were taken at X100 (left) and X400 (right) magnification. Key: green- atretic follicle, yellow-secondary follicle simultaneous development. The magnification used was X100 and X400 and scales of 100 μm and 10 μm, respectively. In **(A)**, the letters above the bar are the same, indicating non-significant difference (*P* > 0.05). *N* = 4, the data are presented as mean ± standard deviation (SD). The number of primordial follicles in each group is as follows: 84.17 ± 8.42^c^, 75.67 ± 7.5^c^, 58.31 ± 11.20^ab^, 47.50 ± 13.97^a^, respectively, *P* < 0.01. The number of growing follicles in each group is as follows: 14.17 ± 2.79^a^, 16.32 ± 5.13^ab^, 23.83 ± 7.35^c^, 20.85 ± 5.78^bc^, respectively, *P* = 0.03. The number of atretic follicles in each group is as follows: 5.01 ± 1.79^a^, 5.67 ± 2.16^a^, 10.53 ± 3.21^b^, 14.36 ± 4.77^c^, respectively, *P* < 0.01. In the same row, values with different small letter superscripts mean significant difference (*P* < 0.05).

The results of the ovarian index are shown in Figure 1A. There was no significant difference between the ovarian index of the prepubertal gilts groups at *P* > 0.05. However, when compared with the CON group, T2 (*P* = 0.058) and T3 (*P* = 0.065) groups exhibited an increasing trend.

### Ovarian structure and morphology

It can be seen from Figures 1B–E that the ovarian capsule of the prepubertal gilts in the CON group was intact, and the number of follicles was normal at all layers of the cortex. The primordial follicles had a small size and were mainly found on the ovarian cortex superficial layer. Besides, the primary follicle size was larger than that of the primordial follicle, while the epithelium follicle had a multilayered cubic or columnar structure with zona pellucida. The secondary follicles cells proliferated between 6 and 12 layers, and the radiation crowns were formed in the follicular cavities and had a few corpus luteum tissues. The medulla connective tissues were loosely arranged and had abundant blood vessels. Compared with the CON group, the number of primordial follicles decreased in all the experimental groups. However, multiple secondary follicles developed simultaneously with increased follicular volume. The number of atretic follicles increased, the corpus luteum was rarely observed, and the medulla connective tissues were loosely arranged. In T2 and T3 experimental groups, the follicles had necrotic and exfoliated granulosa cells with either contracted or fragmented nuclei. These results show that ZEN promotes follicle development, and an increase in dosage increases the effects on the follicles. In addition, ZEN causes follicular atresia, and an increase in dosage significantly increases the number of atretic follicles. The follicles had necrotic and detached granular cells with either contracted or broken nuclei, resulting in ovarian damage. In this study, ZEN did not cause ovary degeneration, necrosis, and hyperplasia. However, in T2 and T3 experimental groups, inflammatory infiltration was observed.

### Serum GHRH content

**Figure 2:**
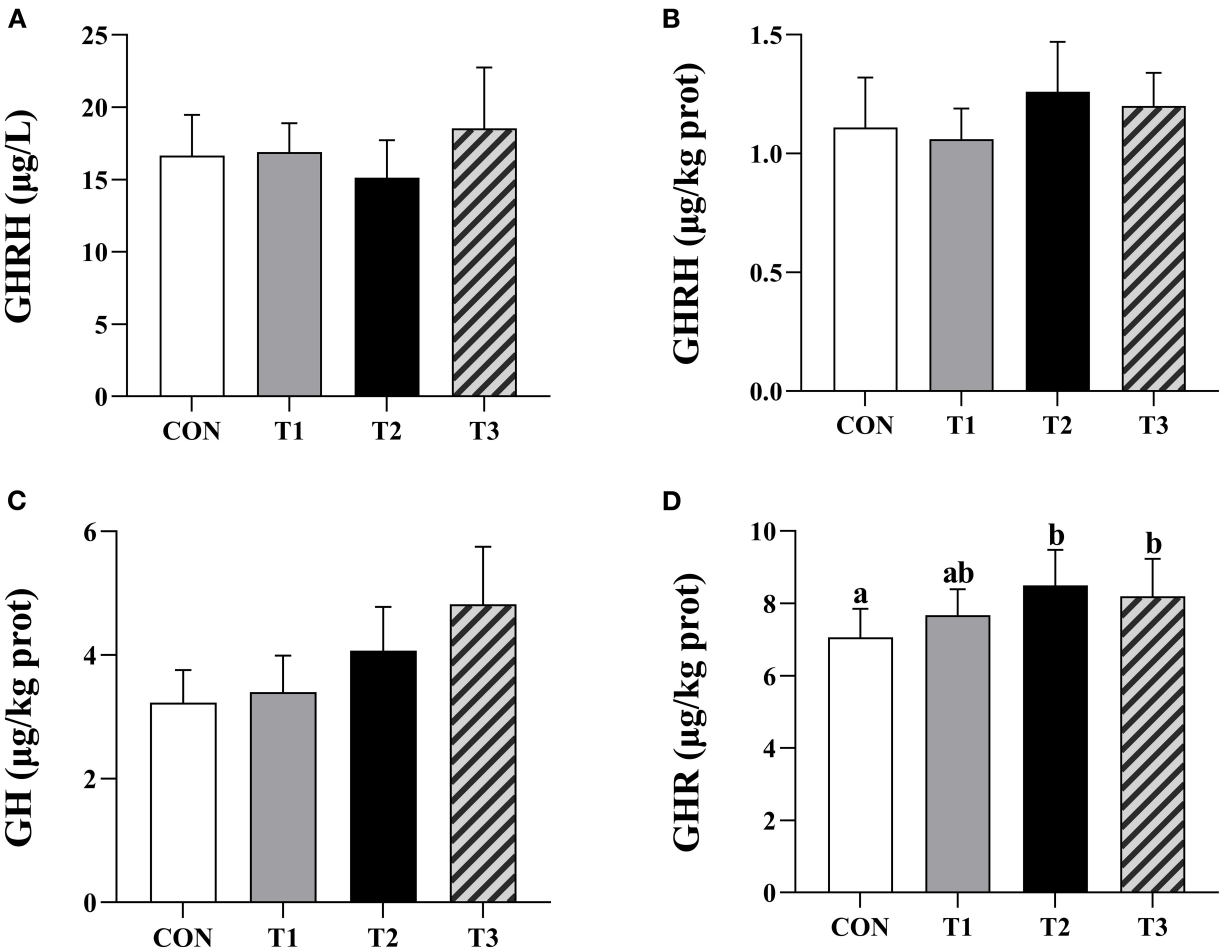
Effects of ZEN on the contents of serum GHRH, hypothalamus GHRH, pituitary GH, and ovarian GHR. Above the bar no letter or the same letter mean no significant (*P* > 0.05), while with different letter mean significant difference (*P* < 0.05). In **(A)**, *N* = 8, in (**B–D)**, *N* = 4, bars represent the means ± standard deviation (SD). Growth hormone-releasing hormone (GHRH), Growth hormone (GH), Growth hormone receptor (GHR). The content of ovarian GHR in each group: 7.06 ± 0.79^a^, 7.67 ± 0.72^ab^, 8.50 ± 0.98^b^, 8.20 ± 1.03^b^, respectively, *P* = 0.03. In the same row, values with different small letter superscripts mean significant difference (*P* < 0.05).

There was no significant difference between the GHRH content of prepubertal gilts serum in each group at *P* > 0.05 (Figure 2A).

### Contents of hypothalamus GHRH, pituitary GH, and ovarian GHR

There was no significant difference in hypothalamic GHRH and pituitary GH contents in each group of the prepubertal gilts at *P* > 0.05 (Figures 2B,C). When compared with the CON group, the ovarian GHR content in T2 and T3 experimental groups was significantly high (*P* < 0.05) (Figure 2D).

### Relative expression levels of hypothalamus *GHRH*, pituitary *GHRHR*, and *GH* mRNA

**Figure 3:**
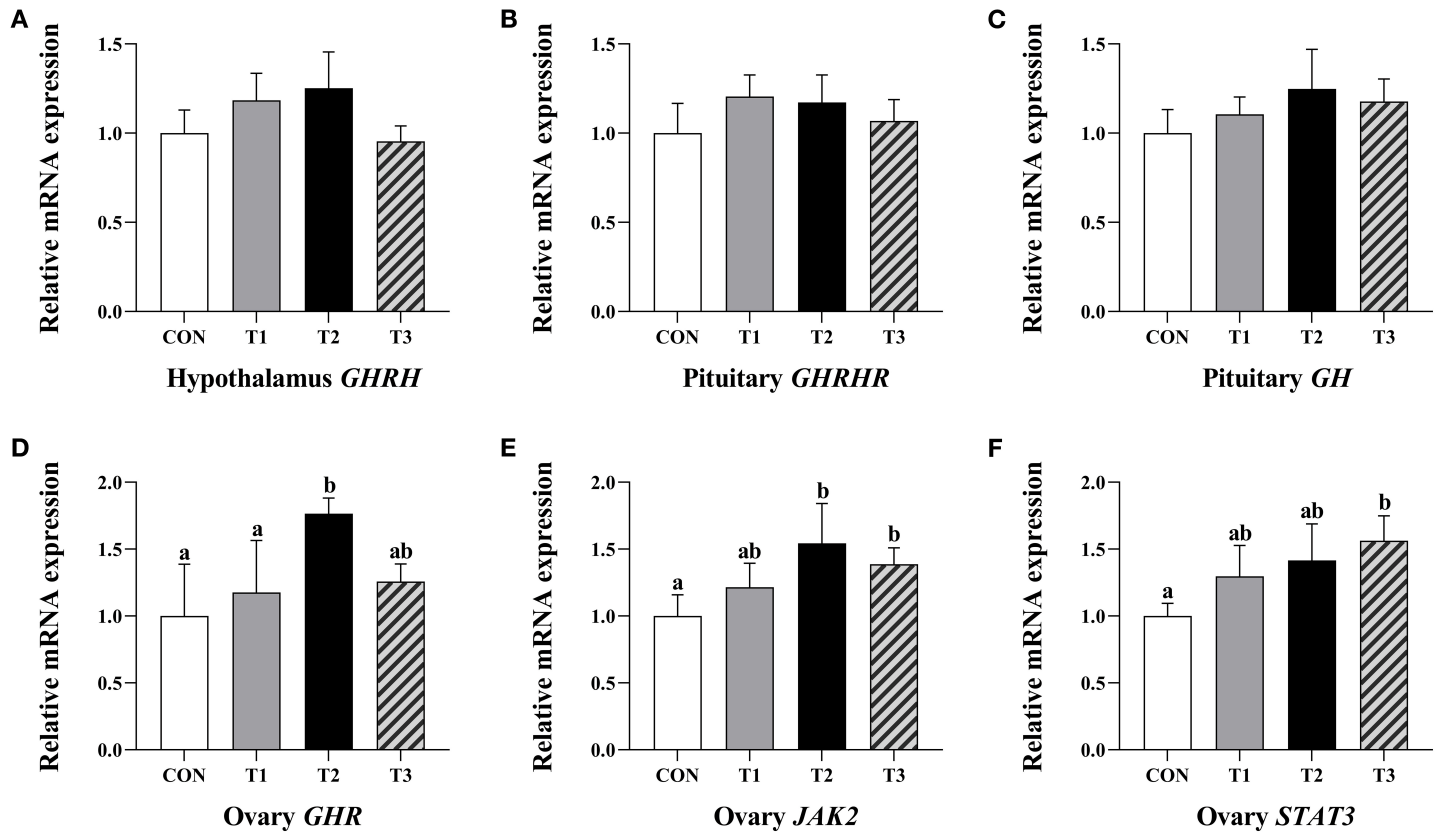
Effects of ZEN on the relative expression level of hypothalamus *GHRH* gene **(A)**, *GHRHR* **(B)** and *GH* gene **(C)** in the pituitary and *GHR* **(D)**, *JAK2* **(E)**, and *STAT3* genes **(F)** in the ovary of prepubertal gilts. The mRNA level of genes were determined by qRT-PCR. Bars represent the means ± standard deviation (SD) (*n* = 12). Above the bar no letter or the same letter mean no significant (*P* > 0.05), while with different letter mean significant difference (*P* < 0.05). Growth hormone-releasing hormone (*GHRH*), Growth hormone-releasing hormone receptor (*GHRHR*), Growth hormone (*GH*), Growth hormone receptor (*GHR*), Janus activated kinase 2 (*JAK2*), Signal transducer and activator of transcriptions 3 (*STAT3*).

There was no significant difference in the relative expression levels of hypothalamus *GHRH*, pituitary *GHRHR*, and *GH* mRNA in each group of the prepubertal gilts at *P* > 0.05 (Figures 3A–C).

### Relative expression level of *GHR, JAK2*, and *STAT3* mRNA in ovary

The T2 group of prepubertal gilts had significantly higher relative expression levels of *GHR* mRNA than the CON and T1 groups (*P* < 0.05). In the T2 group, the relative expression levels of *JAK2* mRNA were significantly high at *P* < 0.05. In the T3 group, the relative expression levels of *JAK2* and *STAT3* mRNA were significantly high at *P* < 0.05 (Figures 3D–F).

### GHR immunoreactive distribution in the prepubertal gilts ovary

**Figure 4:**
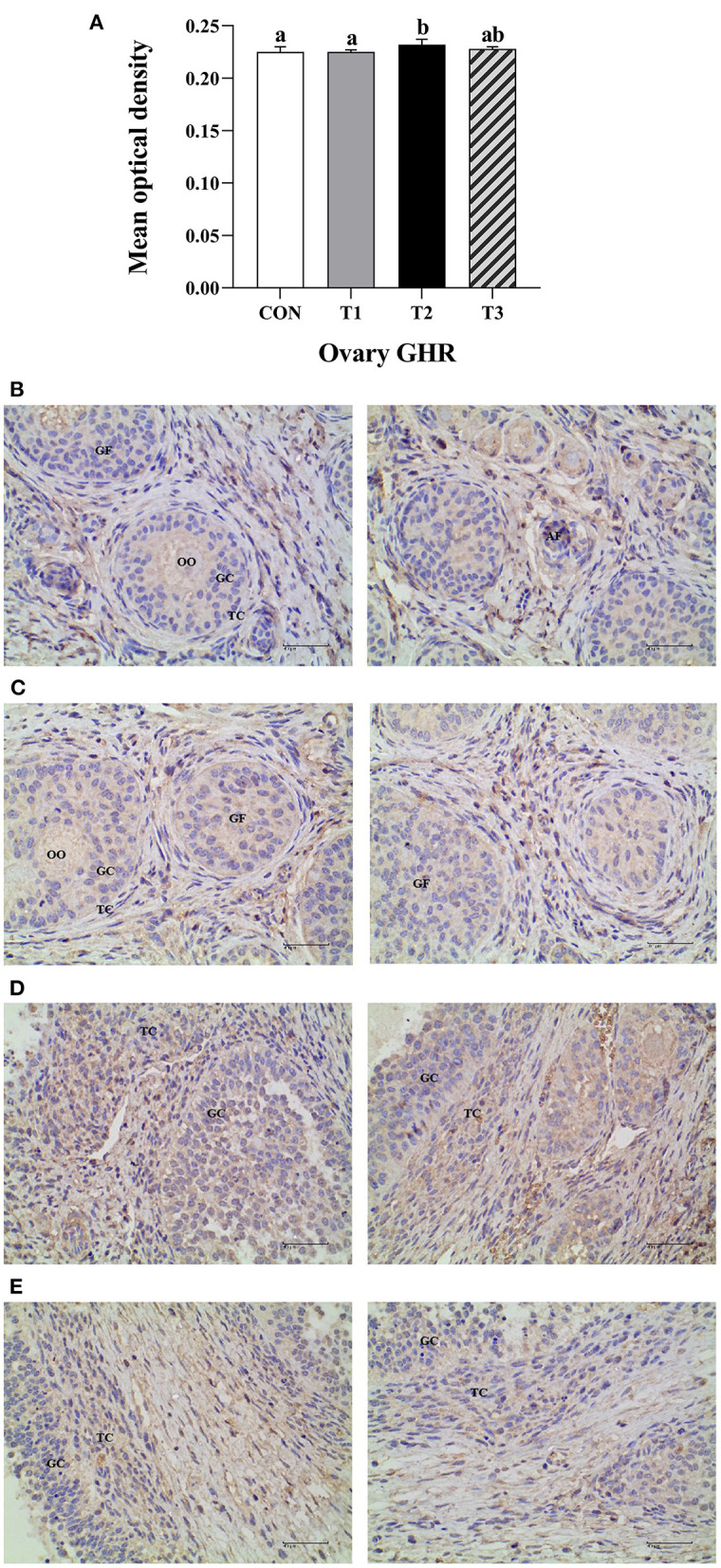
Effects of ZEN on the GHR immunoreactive distribution in the prepubertal gilts ovary. Above the bar no letter or the same letter mean no significant (*P* > 0.05), while with different letter mean significant difference (*P* < 0.05) (*n* = 12) **(A)**. Visual fields of ovarian tissues obtained from the CON **(B)**, T1 **(C)**, T2 **(D)**, and T3 **(E)** experimental groups. All the images were taken at X400 magnification. Key: Primordial follicle (PF), Growing follicle (GF), Atresia follicle (AF), Oocyte (OO), Granulosa cell (GC), Theca cell (TC). The scale bar represents 40 μm.

The average optical density of GHR ovarian tissue in T2 experimental group was significantly higher than that of the CON group and T1 experimental group at *P* < 0.05 (Figure 4A).The immunoreactive substances of GHR were mainly distributed in the ovarian follicles granulosa cells, cytoplasm, and oocytes interstitial cells, while a few were distributed in the theca follicles (Figures 4B–E). The positive cells were either yellow or brownish-yellow. Besides, ZEN had no effect on the GHR distribution and location in the prepubertal gilts ovaries. Compared with the CON group, the T1 experimental group follicle development was accelerated, leading to follicle expansion and increased size. In this study, ZEN increased the ovarian granulosa cell numbers and the immune intensity. The multiple secondary follicles developed simultaneously in the T2 and T3 experimental groups, leading to expansion and increased follicular volume. The number of ovarian granulosa cells further increased, resulting in increased immunoreactive substances. These results show that ZEN promotes ovary and follicle development by increasing the distribution of GHR immunoreactive substances in ovaries and enhances the immunoreactive intensity of the prepubertal gilts.

## Discussion

Comparison of CON group with experimental groups in this experiment showed that the ovarian index in T2 and T3 experimental groups tended to increase while the number of primordial follicles decreased. Besides, multiple secondary follicles developed simultaneously, with an expansion of the follicles and increased follicular volume, indicating that ZEN promotes the development of the ovarian (follicle) in the prepubertal gilts. There have been reports on ZEN ovarian development promoting effects. One of them is Dai et al. (6), who reported that adding 1.04 mg/kg of ZEN into piglet diet promotes ovarian development. In addition, Fu et al. (18), reported that feeding Tibetan piglets with feeds containing 1.13 mg/kg of ZEN promotes the development of the ovary and uterus. Previously, it has been demonstrated that ZEN promotes the development of porcine ovaries and follicles through different ways, such as activating ERs/GSK-3β-dependent Wnt-1/β-catenin signaling pathway Yang et al. (7), promoting autocrine pathway in piglet ovaries, and regulating proliferating cell nuclear antigen (*PCNA*) gene expression (6). Moreover, offspring exposure to ZEN through breast milk affects the development of offspring ovaries (8). This experiment mainly investigated whether ZEN could affect ovarian development through GHRH, GH, and GHR.

Additionally, GHRH is a peptide hormone mainly synthesized by the hypothalamus arcuate nucleus and ventromedial nucleus. This hormone combines with GHRHR in the anterior pituitary and regulates the synthesis and secretion of GH through the cyclic adenosine monophosphate (cAMP) signaling pathway (9). In all the experimental groups, there was no significant difference in GHRH serum content, hypothalamic GHRH content, pituitary GH content, hypothalamic *GHRH* relative expression levels, and pituitary *GHRHR* mRNA. ZEN had no significant effect on the GH content of the gilts serum Wu et al. (19), in line with our previous findings. This implied that ZEN had no significant impact on GH production and secretion in pituitary of prepubertal gilts. Also, Olivares et al. (20) found that zeranol had no significant effect on GH content in lamb serum. However, Thomas et al. (21) reported that zeranol promotes GH secretion through the expression of *Pit-1, GHRHR*, and *GH* mRNA in sheep pituitary. The results from this experiment are contrary to those reported by Thomas et al. (21), and this is attributed to the differences in the experimental materials and experimental animals. Different animals have different sensitivity to ZEN and its metabolites (1).

The GH exerts its function at the tissue and cell level, where it binds with GHR on the target cell membrane surface and dimerizes the GHR. After activation of JAK2, the intracellular structure of GHR and its tyrosine residues are phosphorylated. In addition, the STAT3 is activated, and the phosphorylation of protein factor is induced, and it generates a cascade amplification effect. As a result, the biological signals are transmitted to trigger biological effects in the cells and regulate the growth and physiological function of the ovarian tissue (22, 23). In this experiment, there was a significant increase in relative expression levels of *GHR* and *JAK2* mRNA in the T2 experimental group and *JAK2* and *STAT3* mRNA in the T3 experimental group. Despite the fact that there was no significant difference in the distribution of GHR immunoreactive substances in ovaries of prepubertal gilts in each group, the immunoreactive intensity of GHR in the experimental group presented an increasing trend. In the T2 experimental group, the average optical density of GHR in prepubertal gilts ovaries increased significantly, suggesting that ZEN had no direct effect on the production and secretion of pituitary GH. However, ZEN increases the relative expression levels of *JAK2* and *STAT3* mRNA by improving the expression levels of *GHR* mRNA and protein in the ovaries and activating the downstream JAK-STAT signaling pathway, thus enhances the biological effects of GH and promotes the development of the ovaries (follicles). Song et al. (24) reported that adding 1.0 mg/kg of ZEN in the piglet diet up-regulates *GHR* mRNA and protein expression levels of the piglet ovaries. The content and function of GHR in tissues affects the biological effects of GH. For example, the mutation of the *GHR* gene reduces the biological effects of GH in boys, which leads to short stature in boys (25). Moreover, Feng et al. (26) reported that GH promotes the proliferation of human endometrial glandular cells through the GHR-JAK2-STAT3 pathway. Application of GHR inhibitor AG490 down-regulates the expression of *GHR, JAK2*, and *STAT3* genes, hence reducing the GH biological effects on cell proliferation rate.

In this experiment, the number of atretic follicles in the experimental group was higher than in the CON group. In T2 and T3 experimental groups, the follicles had necrotic and exfoliated granulosa cells with either contracted or fragmented nuclei, which may be associated with the toxic effects of the oxidative stress Xu et al. (27), inflammatory reaction Zhang et al. (28) and autophagy caused by ZEN (29). Abnormal development of ovaries (follicles) and pathological changes in the ovaries increases risks to ovarian hypoplasia, premature aging, and abnormality in ovary functions hence leads to decreased reproductive performance in gilts (30, 31).

## Conclusions

Based on this study, ZEN has no significant effect on the production and secretion of pituitary GH in prepubertal gilts. However, ZEN enhances GH biological effects, promotes the development of the ovary (follicle), and exerts reproductive toxicity by increasing the ovary *GHR, JAK2*, and *STAT3* mRNA expression level and improves the immune intensity of the GHR protein.

## Data availability statement

The original contributions presented in the study are included in the article/supplementary material, further inquiries can be directed to the corresponding author.

## Ethics statement

The animal study was reviewed and approved by the Animal Care and Use Committee of Hebei Agriculture University (Baoding, China). All animal experiments complied with the ARRIVE guidelines were carried out in accordance with the U.K. Animals (Scientific Procedures) Act, 1986 and associated guide-lines, EU Directive 2010/63/EU for animal experiments.

## Author contributions

FW, LG, FL, SC, and BC designed the experiment and wrote the manuscript. FW, LG, and FL performed the statistical analyses. FW, LG, and JC carried out the animal experiment. FW, XY, and YL conducted the sample collection. FW, LG, and FL carried out the sample analysis. All authors contributed to the article and approved the submitted version.

## Funding

This study was supported by Key Research and Development Program of Hebei Province (20326613D), Research Development Fund of Hebei Agricultural University (3003003) and Key Research and Development Program of China (S2016G4513).

## Conflict of interest

The authors declare that the research was conducted in the absence of any commercial or financial relationships that could be construed as a potential conflict of interest.

## Publisher's note

All claims expressed in this article are solely those of the authors and do not necessarily represent those of their affiliated organizations, or those of the publisher, the editors and the reviewers. Any product that may be evaluated in this article, or claim that may be made by its manufacturer, is not guaranteed or endorsed by the publisher.
